# Lopinavir/Ritonavir and Darunavir/Cobicistat in Hospitalized COVID-19 Patients: Findings From the Multicenter Italian CORIST Study

**DOI:** 10.3389/fmed.2021.639970

**Published:** 2021-06-09

**Authors:** Augusto Di Castelnuovo, Simona Costanzo, Andrea Antinori, Nausicaa Berselli, Lorenzo Blandi, Marialaura Bonaccio, Raffaele Bruno, Roberto Cauda, Alessandro Gialluisi, Giovanni Guaraldi, Lorenzo Menicanti, Marco Mennuni, Ilaria My, Agostino Parruti, Giuseppe Patti, Stefano Perlini, Francesca Santilli, Carlo Signorelli, Giulio G. Stefanini, Alessandra Vergori, Walter Ageno, Luca Aiello, Piergiuseppe Agostoni, Samir Al Moghazi, Rosa Arboretti, Filippo Aucella, Greta Barbieri, Martina Barchitta, Alessandro Bartoloni, Carolina Bologna, Paolo Bonfanti, Lucia Caiano, Laura Carrozzi, Antonio Cascio, Giacomo Castiglione, Mauro Chiarito, Arturo Ciccullo, Antonella Cingolani, Francesco Cipollone, Claudia Colomba, Crizia Colombo, Francesco Crosta, Giovanni Dalena, Chiara Dal Pra, Gian Battista Danzi, Damiano D'Ardes, Katleen de Gaetano Donati, Francesco Di Gennaro, Giuseppe Di Tano, Gianpiero D'Offizi, Tommaso Filippini, Francesco Maria Fusco, Carlo Gaudiosi, Ivan Gentile, Giancarlo Gini, Elvira Grandone, Gabriella Guarnieri, Gennaro L. F. Lamanna, Giovanni Larizza, Armando Leone, Veronica Lio, Angela Raffaella Losito, Gloria Maccagni, Stefano Maitan, Sandro Mancarella, Rosa Manuele, Massimo Mapelli, Riccardo Maragna, Lorenzo Marra, Giulio Maresca, Claudia Marotta, Franco Mastroianni, Maria Mazzitelli, Alessandro Mengozzi, Francesco Menichetti, Jovana Milic, Filippo Minutolo, Beatrice Molena, R. Mussinelli, Cristina Mussini, Maria Musso, Anna Odone, Marco Olivieri, Emanuela Pasi, Annalisa Perroni, Francesco Petri, Biagio Pinchera, Carlo A. Pivato, Venerino Poletti, Claudia Ravaglia, Marco Rossato, Marianna Rossi, Anna Sabena, Francesco Salinaro, Vincenzo Sangiovanni, Carlo Sanrocco, Laura Scorzolini, Raffaella Sgariglia, Paola Giustina Simeone, Michele Spinicci, Enrico Maria Trecarichi, Giovanni Veronesi, Roberto Vettor, Andrea Vianello, Marco Vinceti, Elena Visconti, Laura Vocciante, Raffaele De Caterina, Licia Iacoviello, The COVID-19 RISK and Treatments (CORIST) Collaboration

**Affiliations:** ^1^Mediterranea Cardiocentro, Napoli, Italy; ^2^Department of Epidemiology and Prevention, IRCCS Neuromed, Pozzilli, Italy; ^3^UOC Immunodeficienze Virali, National Institute for Infectious Diseases L. Spallanzani, IRCCS, Roma, Italy; ^4^Section of Public Health, Department of Biomedical, Metabolic and Neural Sciences, University of Modena, Modena, Italy; ^5^IRCCS Policlinico San Donato, San Donato Milanese, Italy; ^6^Division of Infectious Diseases I, Fondazione IRCCS Policlinico San Matteo, Pavia, Italy; ^7^Department of Clinical, Surgical, Diagnostic, and Paediatric Sciences, University of Pavia, Pavia, Italy; ^8^Fondazione Policlinico Universitario A. Gemelli IRCCS, Roma, Italy; ^9^Università Cattolica del Sacro Cuore- Dipartimento di Sicurezza e Bioetica Sede di Roma, Roma, Italy; ^10^Infectious Disease Unit, Department of Surgical, Medical, Dental and Morphological Sciences, University of Modena and Reggio Emilia, Modena, Italy; ^11^University of Eastern Piedmont, Maggiore della Carità Hospital, Novara, Italy; ^12^Humanitas Clinical and Research Hospital IRCCS, Rozzano, Italy; ^13^Department of Infectious Disease, Azienda Sanitaria Locale (AUSL) di Pescara, Pescara, Italy; ^14^Emergency Department, IRCCS Policlinico San Matteo Foundation, Pavia, Italy; ^15^Department of Internal Medicine, University of Pavia, Pavia, Italy; ^16^Department of Medicine and Aging, Clinica Medica, SS. Annunziata Hospital and University of Chieti, Chieti, Italy; ^17^School of Medicine, Vita-Salute San Raffaele University, Milano, Italy; ^18^HIV/AIDS Department, National Institute for Infectious Diseases Lazzaro Spallanzani-IRCCS, Roma, Italy; ^19^Department of Medicine and Surgery, University of Insubria, Varese, Italy; ^20^UOC, Anestesia e Rianimazione, Dipartimento di Chirurgia Generale Ospedale Morgagni-Pierantoni, Forlì, Italy; ^21^Centro Cardiologico Monzino IRCCS, Milano, Italy; ^22^Cardiovascular Section, Department of Clinical Sciences and Community Health, University of Milano, Milano, Italy; ^23^UOC Infezioni Sistemiche dell'Immunodepresso, National Institute for Infectious Diseases L. Spallanzani, IRCCS, Rome, Italy; ^24^Department of Civil Environmental and Architectural Engineering, University of Padova, Padova, Italy; ^25^Fondazione IRCCS Casa Sollievo della Sofferenza, San Giovanni Rotondo, Foggia, Italy; ^26^Department of Clinical and Experimental Medicine, Azienda Ospedaliero-Universitaria Pisana, University of Pisa, Pisa, Italy; ^27^Department of Medical and Surgical Sciences and Advanced Technologies G.F. Ingrassia, University of Catania, Catania, Italy; ^28^Department of Experimental and Clinical Medicine, University of Florence and Azienda Ospedaliero-Universitaria Careggi, Firenze, Italy; ^29^Ospedale del Mare, ASL Napoli 1, Napoli, Italy; ^30^UOC Malattie Infettive, Ospedale San Gerardo, ASST Monza, Monza, Italy; ^31^School of Medicine and Surgery, University of Milano-Bicocca, Milano, Italy; ^32^Cardiovascular and Thoracic Department, Azienda Ospedaliero-Universitaria Pisana, University of Pisa, Pisa, Italy; ^33^Infectious and Tropical Diseases Unit- Department of Health Promotion, Mother and Child Care, Internal Medicine and Medical Specialties (PROMISE) - University of Palermo, Palermo, Italy; ^34^Servizio di Anestesia e Rianimazione II UO Rianimazione Ospedale San Marco, AOU Policlinico-Vittorio Emanuele, Catania, Italy; ^35^COVID-19 Unit, EE Ospedale Regionale F. Miulli, Acquaviva delle Fonti, Italy; ^36^Clinica Medica 3, Department of Medicine - DIMED, University Hospital of Padova, Padova, Italy; ^37^Department of Cardiology, Ospedale di Cremona, Cremona, Italy; ^38^Medical Direction, IRCCS Neuromed, Pozzilli, Italy; ^39^UOC Malattie Infettive-Epatologia, National Institute for Infectious Diseases L. Spallanzani, IRCCS, Roma, Italy; ^40^UOC Infezioni Sistemiche e dell'Immunodepresso, Azienda Ospedaliera dei Colli, Ospedale Cotugno, Napoli, Italy; ^41^Ospedale di Boscotrecase - ASL Napoli 3, Napoli, Italy; ^42^Department of Clinical Medicine and Surgery, University of Naples Federico II, Napoli, Italy; ^43^Respiratory Pathophysiology Division, Department of Cardiologic, Thoracic and Vascular Sciences, University of Padova, Padova, Italy; ^44^UOC di Pneumologia, P.O. San Giuseppe Moscati, Taranto, Italy; ^45^ASST Milano Nord - Ospedale Edoardo Bassini Cinisello Balsamo, Milan, Italy; ^46^UOC Malattie Infettive e Tropicali, P.O. San Marco, AOU Policlinico-Vittorio Emanuele, Catania, Italy; ^47^UOC di Medicina - Presidio Ospedaliero S.Maria di Loreto Nuovo, Napoli, Italy; ^48^Infectious and Tropical Diseases Unit, Department of Medical and Surgical Sciences, Magna Graecia University, Catanzaro, Italy; ^49^Dipartimento di Farmacia, Università di Pisa, Pisa, Italy; ^50^UOC Malattie Infettive-Apparato Respiratorio, National Institute for Infectious Diseases L. Spallanzani, IRCCS, Roma, Italy; ^51^Computer Service, University of Molise, Campobasso, Italy; ^52^Medicina Interna. Ospedale di Ravenna, AUSL della Romagna, Ravenna, Italy; ^53^UOC Pneumologia, Dipartimento di Malattie Apparato Respiratorio e Torace, Ospedale Morgagni-Pierantoni, Forlì, Italy; ^54^UOC Malattie Infettive ad Alta Intensità di Cura, National Institute for Infectious Diseases L. Spallanzani, IRCCS, Rome, Italy; ^55^Department of Epidemiology, Boston University School of Public Health, Boston, MA, United States

**Keywords:** COVID-19, SARS-CoV-2, darunavir, lopinavir, in-hospital mortality

## Abstract

**Background:** Protease inhibitors have been considered as possible therapeutic agents for COVID-19 patients.

**Objectives:** To describe the association between lopinavir/ritonavir (LPV/r) or darunavir/cobicistat (DRV/c) use and in-hospital mortality in COVID-19 patients.

**Study Design:** Multicenter observational study of COVID-19 patients admitted in 33 Italian hospitals. Medications, preexisting conditions, clinical measures, and outcomes were extracted from medical records. Patients were retrospectively divided in three groups, according to use of LPV/r, DRV/c or none of them. Primary outcome in a time-to event analysis was death. We used Cox proportional-hazards models with inverse probability of treatment weighting by multinomial propensity scores.

**Results:** Out of 3,451 patients, 33.3% LPV/r and 13.9% received DRV/c. Patients receiving LPV/r or DRV/c were more likely younger, men, had higher C-reactive protein levels while less likely had hypertension, cardiovascular, pulmonary or kidney disease. After adjustment for propensity scores, LPV/r use was not associated with mortality (HR = 0.94, 95% CI 0.78 to 1.13), whereas treatment with DRV/c was associated with a higher death risk (HR = 1.89, 1.53 to 2.34, E-value = 2.43). This increased risk was more marked in women, in elderly, in patients with higher severity of COVID-19 and in patients receiving other COVID-19 drugs.

**Conclusions:** In a large cohort of Italian patients hospitalized for COVID-19 in a real-life setting, the use of LPV/r treatment did not change death rate, while DRV/c was associated with increased mortality. Within the limits of an observational study, these data do not support the use of LPV/r or DRV/c in COVID-19 patients.

## Introduction

After more than 1 year of COVID-19 pandemic there are still no solid certainties on the efficacy of the therapies variously proposed. The urgency to intervene has induced drug agencies to allow the use of off-label drugs, although only few clinical trials have already been published.

Protease inhibitors have been considered as a candidate therapy because they inhibit enzymes that activate envelope glycoproteins as part of the process of viral entry into cells (1). Lopinavir is a human immunodeficiency virus (HIV) type-1 aspartate protease inhibitor, with an *in vitro* inhibitory activity against the coronaviruses causing severe acute respiratory syndrome (SARS) (2) and Middle-East respiratory syndrome (MERS) (3). It is administered in combination with ritonavir to increase its plasma half-life. Both drugs have been shown to be able to bind well to the SARS-CoV 3C-like protease (3CLpro) (4), which is involved in the proteolytic processing of the replicase polyprotein and is crucial for viral replication (5). However, the efficacy of this combination in patients with SARS or MERS was based on scarce data (6).

Both the Recovery (7) and the Solidarity trial (8) failed to observe any clinical benefit of lopinavir/ritonavir (LPV/r) treatment beyond standard care in hospitalized patients with severe COVID-19. Null efficacy of LPV/r was also observed in other clinical trials (9) or retrospective studies, as systematically reviewed (10).

Given the structural similarity with lopinavir, darunavir, another protease inhibitor used in HIV therapy (11, 12), with cobicistat as a pharmaco-enhancer, has also been proposed as a COVID-19 treatment (13). In the emergency phase of COVID-19 pandemic the Italian Drug Agency (AIFA) (14) allowed the therapeutic use of both LPV/r and darunavir/cobicistat (DRV/c). However, evidence for the efficacy of DRV/c in COVID-19 patients is scarce, and findings from randomized clinical trials are lacking. In this context of uncertainty, sufficiently powered retrospective observational studies may be useful to shed light on the efficacy of these drugs in the SARS-CoV-2 pandemic.

We analyzed the association between DRV/c or LPV/r use and mortality in 3,451 COVID-19 patients from 33 clinical centers all over Italy.

## Materials and Methods

### Setting

This national retrospective observational study was conceived within the CORIST Project (ClinicalTrials.gov ID: NCT04318418), which is a multicenter study launched in March 2020 (15) and aimed at testing the association of risk factors (16) and therapies with in-hospital COVID-19 mortality (17, 18). The study was approved by the institutional ethics board of all recruiting centers. Data for the present analyses were provided by 33 hospitals distributed throughout Italy (Appendix). Each hospital provided data from hospitalized patients (≥18 years of age) who had a positive test result for the SARS-CoV-2 virus at any time during their hospitalization from February 19 to May 23, 2020. The follow-up continued through May 29, 2020.

### Data Sources

We obtained data from a cohort comprising 3,971 COVID-19 patients. The SARS-CoV-2 status was based on polymerase chain reaction on nasopharyngeal swab. Data were extracted at one-time point from electronic medical records or charts. Data included patients' demographics, laboratory tests, historical and current medication lists and diagnoses. Information on the most severe manifestation of COVID-19 occurred during hospitalization was retrospectively captured (16). We obtained the following information for each patient: date of admission and date of discharge or death; age; sex; the first recorded laboratory tests at entry; past comorbidities (coronary disease, diabetes, hypertension, respiratory disease and cancer) and current drug therapies for COVID-19—DRV/c, LPV/r, hydroxychloroquine (HCQ), remdesivir, tocilizumab, sarilumab, corticosteroids. Chronic kidney disease was classified by using of glomerular filtration rate (GFR) as reported in footnote of Table 1. Patients were defined as receiving LPV/r or DRV/c if they were receiving it at admission to hospital or received it during the follow-up period. Every physician in each hospital decided for him or herself if and how to treat their patient. According to the AIFA guidance (13, 14), LPV/r was administered at the dose of 400/100 mg ×2/day and DRV/c at the dose of 800/150 mg/day, both for at least 5–7 days, according to the clinical evolution of disease.

**Table 1:** General characteristics of COVID-19 patients at baseline, according to lopinavir/ritonavir (LPV/r) or darunavir/cobicistat (DRV/c) use.

**Characteristic**	Controls^*^(**N** = 1, 824)	**LPV/r (*N* = 1,148)**	**DRV/c (*N* = 479)**	***P*-value unadjusted^^^**	***P*-value adjusted^**#**^**
**Age-median (IQR-yr.)**	69 (56–80)	65 (55–76)	65 (58–77)	<0.0001	0.65
**Gender-no (%)**				<0.0001	0.65
Women	774 (42.4%)	386 (33.6%)	141 (29.4%)		
Men	1,050 (57.6%)	762 (66.4%)	338 (70.6%)		
**Diabetes-no (%)**^**‡**^				0.12	0.91
No	1,422 (79.0%)	937 (82.0%)	364 (79.3%)		
Yes	377 (21.0%)	205 (18.0%)	95 (20.7%)		
**Hypertension-no (%)**^**‡**^				0.0068	0.63
No	853 (47.4%)	564 (49.3%)	255 (55.7%)		
Yes	946 (52.6%)	579 (50.7%)	203 (44.3%)		
**Ischemic heart disease-no (%)**^**‡**^				0.0046	0.76
No	1,449 (80.9%)	962 (84.8%)	389 (86.1%)		
Yes	341 (19.1%)	173 (15.2%)	63 (13.9%)		
**Chronic pulmonary disease-no (%)**^**‡**^				0.0003	0.64
No	1,489 (83.1%)	1,000 (87.7%)	402 (88.6%)		
Yes	304 (16.9%)	140 (12.3%)	52 (11.4%)		
**Cancer-no (%)**^**‡**^				0.17	0.75
No	1,590 (88.4%)	1,034 (90.6%)	408 (89.5%)		
Yes	208 (11.6%)	107 (9.4%)	48 (10.5%)		
**CKD stage**^¶^**-no (%)**^**‡**^				<0.0001	0.57
Stage 1	629 (35.4%)	399 (35.2%)	183 (39.0%)		
Stage 2	615 (34.6%)	490 (43.2%)	167 (35.6%)		
Stage 3a or stage 3b	377 (21.2%)	202 (17.8%)	88 (18.8%)		
Stage 4 or stage 5	158 (8.9%)	43 (3.4%)	31 (6.6%)		
**C Reactive Protein-no (%)**^**‡**^				<0.0001	0.10
<1 mg/L	235 (13.7%)	95 (8.7%)	30 (6.4%)		
1-3 mg/L	215 (12.6%)	153 (14.0%)	53 (11.4%)		
>3 mg/L	1,263 (73.7%)	845 (77.3%)	384 (82.2%)		
**Hydroxychloroquine use**				<0.0001	0.18
No	621 (34.0%)	170 (14.8%)	26 (5.4%)		
Yes	1,203 (66.0%)	978 (85.2%)	453 (94.6%)		
**Tocilizumab or Sarilumab use**				0.38	0.93
No	1,526 (83.7%)	981 (85.4%)	408 (85.2%)		
Yes	298 (16.3%)	167 (14.6%)	71 (14.8%)		
**Remdesivir use**				0.35	0.19
No	1,781 (97.6%)	1,111 (96.8%)	467 (97.5%)		
Yes	43 (2.4%)	37 (3.2%)	12 (2.5%)		
**Corticosteroids use**				0.11	0.25
No	1,163 (63.8%)	775 (67.5%)	313 (65.3%)		
Yes	661 (36.2%)	373 (32.5%)	166 (34.7%)		
**Clusters of hospitals**				<0.0001	0.19
Northern regions (except Milan) (n)	414 (22.7%)	268 (23.3%)	103 (21.5%)		
Milan (m)	340 (18.6%)	224 (19.5%)	122 (25.5%)		
Center regions (except Rome) (c)	674 (36.9%)	186 (16.2%)	190 (39.7%)		
Rome (r)	109 (6.0%)	321 (28.0%)	54 (11.3%)		
Southern regions (s)	287 (15.7%)	149 (13.0%)	10 (2.1%)		

* *Control group was formed by patients with neither LPV/r nor DRV/c.*

^ *Chi-square test.*

# *Adjusted by inverse probability by treatment weighting as obtained by multinomial propensity score.*

‡ *Missing values were N = 51 for diabetes, N = 51 for hypertension, N = 74 for ischemic heart disease, N = 64 for chronic pulmonary disease, N = 56 for cancer, N = 69 for CKD stage and N = 178 for C reactive protein.*

¶ *Stage 1: Kidney damage with normal or increased glomerular filtration rate (GFR) (>90mL/min/1.73m^2^); Stage 2: Mild reduction in GFR (60–89 mL/min/1.73 m^2^); Stage 3a: Moderate reduction in GFR (45–59 mL/min/1.73 m^2^); Stage 3b: Moderate reduction in GFR (30–44 mL/min/1.73 m^2^); Stage 4: Severe reduction in GFR (15–29 mL/min/1.73 m^2^); Stage 5: Kidney failure (GFR <15mL/min/1.73m^2^ordialysis). GFR was calculated by the Chronic Kidney Disease Epidemiology Collaboration (CKD-Epi) equation. (n) includes hospitals of 5–10; (m) includes hospitals 1–4; (c) includes hospitals 11–17; (r) includes hospitals 18–20; (s) includes hospitals 21–33 (see Appendix)*.

### Statistical Analyses

The study index date was defined as the date of hospital admission. Index dates ranged from February 19, 2020 to May 23, 2020. The study end point was the time from study index to death. The number of patients who either died, or had been discharged alive, or were still admitted to hospital as of May 29, 2020, were recorded, and hospital length of stay was determined. Patients alive had their data censored on the date of discharge. Data were censored at 35 days in *N* = 330 (8.3%) patients with a follow up >35 days.

Of the initial cohort of 3,971 patients, 350 patients were excluded from the analysis because of missing data on LPV/r or DRV/c use (*N* = 112), other drug COVID-19 therapies (hydroxychloroquine, tocilizumab or sarilumab, remdesivir or corticosteroids, *N* = 247), time to event (*N* = 59), outcome (*N* = 8), COVID-19 severity (*N* = 4), age (*N* = 4), or sex (*N* = 2). Of the remaining 3,621 patients, 170 patients died or were discharged within 24 h after presentation, and were also excluded from the analysis.

At the end, the analyzed cohort consisted of *N* = 3,451 patients. Among them, 8.5% had at least a missing value for covariates. Distribution of missing values was as follows: C-reactive protein (*N* = 178); GFR (*N* = 69); ischemic disease (*N* = 74); chronic pulmonary disease (*N* = 64); diabetes (*N* = 51); hypertension (*N* = 51); and cancer (*N* = 56). We used multiple imputation techniques (*N* = 10 imputed datasets) to maximize data availability. We also conducted a case-complete analysis on 3,156 patients.

Cox proportional-hazards regression models were used to estimate the association between drugs use and death. Since multiple imputation was applied, the final standard error was obtained using the Rubin's rule (19). The proportional hazards assumption was assessed using weighed Schoenfeld residuals, and no violation was identified. To account for the non-randomized drugs administration, we used the multinomial propensity-score method (20). Individual propensities for receiving LPV/r or DRV/c treatment were assessed with the use of a multivariable logistic-regression model based on the generalized logit and including age, sex, diabetes, hypertension, history of ischemic heart disease, chronic pulmonary disease, GFR, C-reactive protein, use of hydroxychloroquine, tocilizumab or sarilumab, remdesivir or corticosteroids and hospitals clustering. Associations between drug treatments and death were then appraised by multivariable Cox regression models with the use of propensity-score and further controlling for hospitals clustering as random effect [frailty model (21)]. The primary analysis used inverse probability by treatment weighting (22). Secondary analyses used multivariable Cox regression analysis or multivariable logistic regression analyses, or accounted for hospitals clustering via stratification or by robust sandwich estimator. Hospitals were clustered according to their geographical distribution, as illustrated in Table 1. To quantify the potential for an unmeasured confounder to render apparent statistically significant hazard ratio non-significant, the E-value was calculated (23). Analyses were performed with the aid of the SAS version 9.4 statistical software for Windows.

## Results

We included in the final analyses 3,451 COVID-19 patients; of these, 1,824 (52.9%, range among hospitals 22.5–64.3%) received neither LPV/r nor DRV/c, 1,148 (33.3%, range 17.7–66.3%) received LPV/r and 479 (13.9%, range 2.2–18.1%) received DRV/c. For both drugs, treatment started as soon as possible after diagnosis confirmation and was 7–15 days long. Half of patients were hospitalized before 22 March 2020. In this first period, the prevalence of patients who received or not LPV/r or DRV/c was 38.5% (neither LPV/r nor DRV/c), 42.7% (LPV/r) and 18.8% (DRV/c). In the second period, the use of protease inhibitors clearly decreased (prevalence became 67.3, 23.8, 8.9%, respectively). However, among patients who received protease inhibitors, the percentage of individuals who were allocated to DRV/c unchanged in the two periods (30.6 and 27.4%, in the first and in the second period, respectively).

Baseline characteristics of the 3 groups are shown in Table 1. Patients receiving LPV/r or DRV/c were more likely younger, men, had higher C-reactive protein but less likely had hypertension, ischemic heart or chronic pulmonary or severe kidney disease. Patients in the LPV/r or DRV/c group more likely received hydroxychloroquine. As expected, all the pre-treatment differences disappeared after adjustment by propensity score weighting (Table 1, c-statistic = 0.72). Percentage of patients who needed of intensive care was 9.5% (in the group with neither LPV/r nor DRV/c), 13.9% (LPV/r) and 10.5% (DRV/c), *P* = 0.0010 for difference.

### Primary Outcome

Out of 3,222 patients, 486 died (15.1%), 2,269 were discharged alive (70.4%) and 467 (14.5%) were still at the hospital. The median follow-up was 14 days (interquartile range 8–23). Death rate (per 1,000 person-days) was 8.2, 15.1, and 10.8 in LPV/r, DRV/c and control group, respectively (Table 2).

**Table 2:** Incidence rates and hazard ratios for death in COVID-19 patients, according to lopinavir/ritonavir (LPV/r) or darunavir/cobicistat (DRV/c) use.

**Multiple Imputation analysis (*N =* 3,451)**	**Death** **(*N =* 576)**	**Patient at risk(*N =* 3,451)**	**Person-days**	**Death Rate (x1,000 person-days)**
Controls (neither LPV/r nor DRV/c)- no. (%)	319 (17.5%)	1,824 (100%)	29,665	10.8
LPV/r- no. (%)	158 (13.8%)	1,148 (100%)	19,172	8.2
DRV/c- no. (%)	99 (20.7%)	479 (100%)	6,551	15.1
**Hazard ratio for death** (Cox regression analysis)			**LPV/r vs. controls HR (95% CI)**	**DRV/c vs. controls HR (95% CI)**
Crude analysis			0.76 (0.62 to 0.91)	1.35 (1.07 to 1.69)
Multivariable analysis^*^			1.12 (0.91 to 1.37)	1.67 (1.31 to 2.14)
Propensity score analysis, inverse probability weighting^**^ **(primary analysis)**			**0.94 (0.78 to 1.13)**	**1.89 (1.53 to 2.34)**
**Odds ratio for death** (logistic regression analysis)			**OR (95% CI)**	**OR (95% CI)**
Propensity score analysis, inverse probability weighting^**^			1.04 (0.85 to 1.27)	1.87 (1.47 to 2.38)
**Case Complete analysis (*****N=*****3,156)**	**Death** (***N=*****510)**	**Patient at risk** (***N=*****3,156)**	**Person-days**	**Death Rate (x1,000 person-days)**
Controls (neither LPV/r nor DRV/c)-no. (%)	286 (17.3%)	1,657 (100%)	28,380	10.1
LPV/r-no. (%)	146 (13.7%)	1,063 (100%)	18,776	7.8
DRV/c- no. (%)	78 (17.9%)	436 (100%)	6,275	12.4
**Hazard ratio for death** (Cox regression analysis)			**LPV/r vs. controls HR (95% CI)**	**DRV/c vs. controls HR (95% CI)**
Crude analysis			0.75 (0.61 to 0.92)	1.14 (0.89 to 1.47)
Multivariable analysis^*^			1.11 (0.89 to 1.37)	1.44 (1.10 to 1.89)
Propensity score analysis, inverse probability weighting^**^			0.94 (0.77 to 1.13)	1.86 (1.49 to 2.32)
**Odds ratio for death** (logistic regression analysis)			**OR (95% CI)**	**OR (95% CI)**
Propensity score analysis, inverse probability weighting^**^			1.05 (0.85 to 1.29)	1.82 (1.41 to 2.34)

*HR, hazard ratios; CI, confidence intervals.*

* *Controlling for age, sex, diabetes, hypertension, history of ischemic heart disease, chronic pulmonary disease, chronic kidney disease, C-reactive protein and use of hydroxychloroquine, tocilizumab or sarilumab, Remdesivir or corticosteroids as fixed effects and hospitals clustering as random effect.*

** *Including hospitals clustering as random effect covariate*.

**Figure 1:**
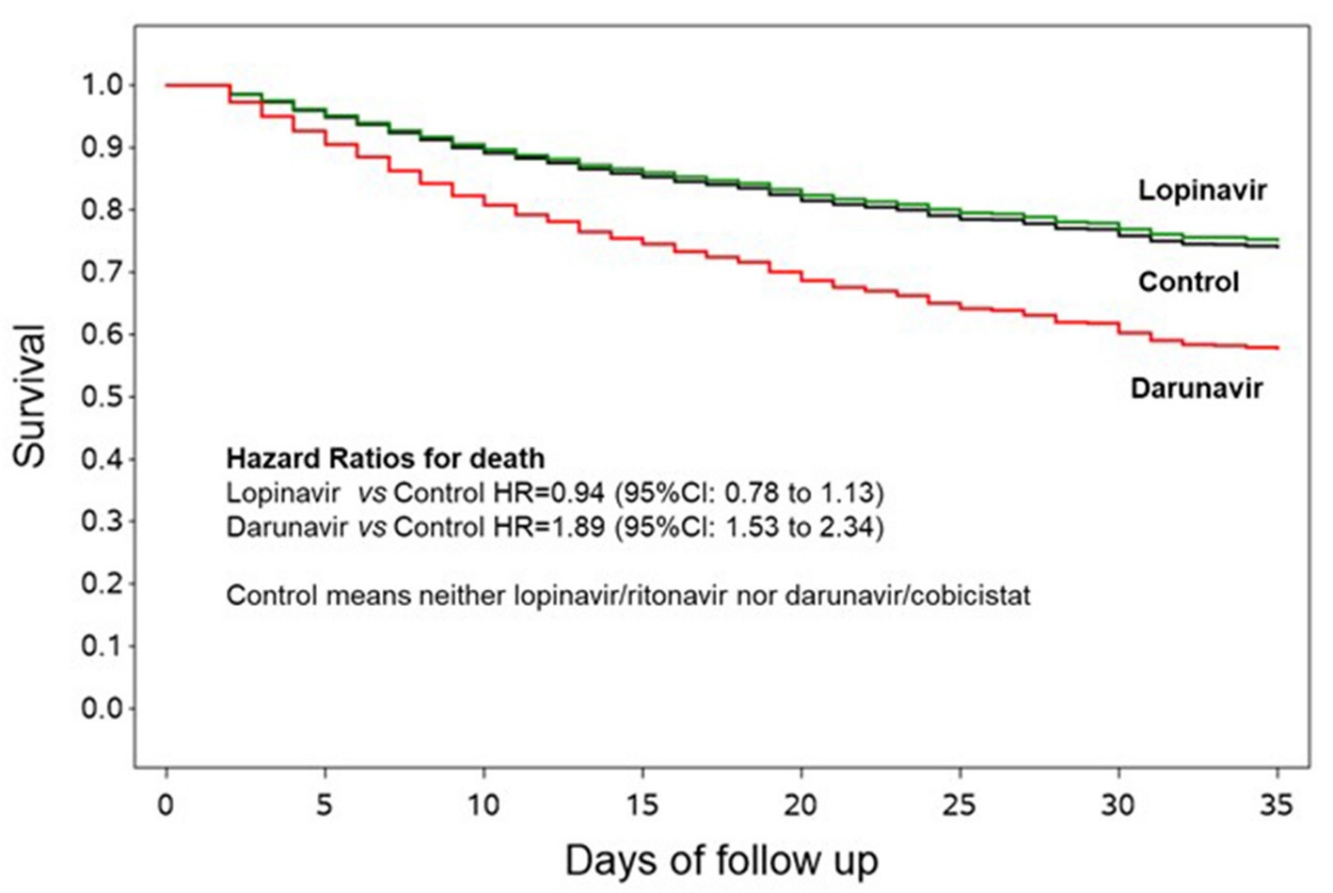
Survival curves according to lopinavir/ritonavir or darunavir/cobicistat use. The curves are adjusted by propensity score analysis (inverse probability by treatment weighting) and hospitals clustering as random effect, and are generated using the first imputed dataset. The other imputed datasets are similar and thus omitted.

As compared to control group, univariable hazard ratios for death were 0.76 (95% CI: 0.62–0.91) and 1.35 (95% CI: 1.07–1.69) for LPV/r and DRV/c, respectively (Table 2). The association with mortality for the LPV/r group disappeared in multivariable analysis (HR = 0.94, 95% CI 0.78–1.13). (Figure 1, Table 2). On the contrary, the increased risk of death associated with DRV/c was reinforced in primary analysis (HR = 1.89, 95% CI 1.53–2.34, E-value for confidence interval = 2.43) (Figure 1, Table 2).

Secondary multivariable analyses yielded very similar results (Table 2), as also happened for the case-complete analyses restricted to the 3,156 patients without missing data or when the association with death was quantified by logistic regression analysis (Table 2).

Control of hospitals clustering with different approaches also yielded similar results (LPV/r group HR = 0.94, 95% CI: 0.78–1.14 and DRV/c group HR = 1.93, 95% CI: 1.55–2.38 when hospitals clustering was stratified for and LPV/r group HR = 0.95, 95% CI: 0.71–1.29 and DRV/c group HR = 1.84, 95% CI: 1.28–2.65 with the robust sandwich estimator). Considering secondary multivariable analyses overall, HR for mortality associated with LPV/r ranged between 0.94 and 1.12, and that associated with DRV/c ranged between 1.44 and 1.93.

Sensitivity analyses are presented in Table 3. LPV/r treatment was not associated with mortality in any subgroup, with the exception of patients with less severe COVID-19 (this finding is plagued by very large uncertainty due to small sample size) and in patients not treated with other anti COVID-19 drugs. The increased mortality risk associated with use of DRV/c was more marked in women, in elderly, in patients with higher severity of COVID-19 and in patients treated for other COVID-19 drugs.

**Table 3:** Hazard ratios for mortality according to lopinavir/ritonavir (LPV/r) or darunavir/cobicistat (DRV/c) use, in different subgroups.

	Controls^*^ **(*N =* 817)**	**LPV/r (*N =* 2,634)**	**DRV/c (*N =* 450)**	**HR (95% CI)**^¶^	
**Subgroups**	**No. death /patient at risk**	**No. death /patient at risk**	**No. death /patient at risk**	**Lopinavir vs. controls**	**Darunavir vs. controls**
Women	124/774	47/386	25/141	0.88 (0.63 to 1.21)	2.41 (1.69 to 3.42)
Men	195/1,050	111/762	74/338	0.96 (0.77 to 1.21)	1.55 (1.18 to 2.03)
Age <70 years	57/922	38/693	20/284	0.86 (0.57 to 1.29)	1.39 (0.84 to 2.33)
Age ≥70 years	262/902	120/455	79/195	1.04 (0.84 to 1.28)	2.20 (1.73 to 2.79)
**Highest degree of COVID-19 severity experienced at hospital**					
Mild pneumonia or less	53/962	13/616	2/204	0.52 (0.29 to 0.92)	0.30 (0.08 to 1.16)
Severe pneumonia	133/514	72/327	47/176	0.94 (0.72 to 1.24)	1.26 (0.89 to 1.77)
Acute respiratory distress syndrome	133/348	73/205	50/99	1.07 (0.80 to 1.42)	2.09 (1.50 to 2.89)
**Use of hydroxychloroquine**					
No	149/621	35/170	6/26	0.58 (0.39 to 0.86)	1.50 (0.99 to 2.29)
Yes	170/1,203	123/978	93/453	0.97 (0.77 to 1.22)	2.02 (1.57 to 2.61)
**Use of other COVID-19 treatments^**					
No	101/439	20/104	3/17	0.50 (0.31 to 0.82)	1.25 (0.74 to 2.12)
Yes	218/1,385	138/1,044	96/462	0.99 (0.80 to 1.22)	1.99 (1.57 to 2.53)

* *Control group was formed by patients with neither LPV/r nor DRV/c. HR, hazard ratios; CI, confidence intervals;*

¶ *Propensity score analysis, inverse probability weighting, including hospital clustering as random effect covariate; multiple imputed analysis. ^tocilizumab or sarilumab or remdesivir or corticosteroids*.

## Discussion

In a large cohort of 3,451 patients hospitalized for COVID-19 in 33 clinical centers all over Italy, treatment with LPV/r did not modify the risk of death, while administration of DRV/c was associated with an increased risk.

Though taking into consideration the limitations of the observational design of our study, our results do not support the use of LPV/r or DRV/c in patients with COVID-19.

Concerning LPV/r use, our findings are in agreement with findings from clinical trials (7–9, 24) and with results of a systematic review pooling data on 6 clinical trials and 10 observational studies (10). In our study, LPV/r was given according to Italian official guidelines, mostly to less severe patients and as early as possible after hospital admission. We performed a series of sensitivity analyses, all confirming the absence of association between LPV/r and risk of death.

In our study DRV/c was associated with a mean 89% increased risk of death, particularly in women, older people, more severely affected or HCQ treated patients, probably due to an increased cardiotoxicity of the drug in these conditions (25).

Although Lopinavir and Darunavir are no longer the gold standard of HIV therapy, their efficacy and safety profile has been well-established in HIV infected patients (11, 26, 27), while there are no clear evidence supporting their use in other viral diseases (28, 29). In fact, the target enzymes involved by HIV and SARS-CoV-2 are quite different: HIV protease is an aspartic protease, whereas SARS-CoV-2 3C-like proteinase is a cysteine protease. Unfortunately, no X-ray crystal structures of 3CLpro complexes including either lopinavir or darunavir are available. Nevertheless, a limited series of computational studies have so far produced contrasting results. In some articles lopinavir was found to have a higher theoretical affinity for SARS-CoV-2 3CLpro than that of darunavir (30, 31). Other articles, instead, describe darunavir as showing large binding free energies to SARS-CoV-2 3CLpro (32–34). These contrasting computational results do not definitely establish whether lopinavir or darunavir is more or less active on the specific SARS-CoV-2 main protease. Nevertheless, when compared to their original indication, both compounds are likely to behave quite differently in the treatment of COVID-19 patients and also to display dissimilar side effects.

Use of DRV/c in COVID-19 patients has been associated with severe drug-drug interactions with concomitant medications that may contribute to death (35). Interestingly, we found an increased relative risk for death associated with DRV/c in older patients (more likely taking other drugs), in patients who experienced at hospital highest degree of COVID-19 severity (more likely taking other drugs) and in patients taking hydroxychloroquine, tocilizumab, sarilumab, remdesivir, or corticosteroids.

Moreover, in spite of the fact that both LPV/r and DRV/c include CYP3A4 inhibitors (ritonavir and cobicistat, respectively) with similar *in vitro* inhibition potencies and subtype selectivities (36), they present remarkable differences in their overall DDI profiles (37); these differences are quite difficult to be placed in a rational correlation with the final clinical outcome, but they should be acknowledged as a possible factor explaining different results in total mortality when comparing LPV/r and DRV/c, as obtained in our study. Furthermore, serious concerns about the possibility that cobicistat, in particular, could produce relevant undesired DDIs were recently raised in analyzing drug combinations for the treatment of HIV infection (38). As already mentioned, lopinavir was found to have a higher theoretical affinity for SARS-CoV-2 3CLpro than darunavir. Therefore, theoretically, lopinavir efficacy might be greater. On the other hand, both drugs have side effects. It is possible that efficacy and side effects balanced for lopinavir (giving a null net effect on mortality) but not for darunavir (giving a net negative effect). Of interest, in an Italian cohort of 689 COVID-19 hospitalized patients followed for negative outcomes (39), it was found that the incidence of in-hospital pulmonary embolism was higher in patients using DRV/c but not LPV/r. On the contrary, other studies did not find an increased rate of severe adverse effects associated with DRV/c (28, 40).

We cannot exclude that patients on DRV/c had a more advanced disease (and then a higher risk of mortality) because DRV/c was used after the run out/shortage of LPV/r. However, we found that among patients who received protease inhibitors (LPV/r or DRV/c), the proportion of individuals who were allocated to DRV/c unchanged during recruitment (from February 2020 to May 2020). This finding suggest that, at least in the CORIST Collaboration, is unlikely that allocation of patients to DRV/c was temporarily biased.

## Strengths and Limitations

A major strength of this study is the large, unselected, real-life patient sample from 33 hospitals, covering the entire Italian territory. This study has, however, several recognized limitations. First of all, we are well aware of the limits of an observational study. However, the CORIST Collaboration was launched at the very beginning of the pandemic, when the general situation in Italy was dramatic and the organization of a controlled clinical trial was considered to be quite difficult. In the absence of any solid data, a prompt, real-life observational study appeared to be the best option at that moment. We took a number of precautions to account for the non-randomized drugs administration procedure and to reduce the effects of confounders by using a propensity-score method. Due to the critical conditions in which the project was launched and the retrospective nature of the study, some parameters were not available in all patients, and not all in-hospital medications and clinical conditions have been recorded. As a consequence, a fully evaluation of disease severity at entry in hospital has not been possible. This is mainly due to our decision to interfere in a quite soft way with the dramatic clinical situation present in the majority of participating hospitals by proposing a relatively simple protocol, asking to report an essential data set information. Use of LPV/r or DRV/c was a missing data for only 2.8% of the whole cohort. For differing reasons, timing of the first dose of LPV/r or DRV/c after presentation to the hospital and duration of treatment could not be provided at individual level by some clinical centers. Although guidelines on the use of LPV/r and DRV/c in COVID-19 patients had been published in Italy since the first phase of the pandemic, individual centers could have deviated from recommendations and used different doses or treatment schemes. Reason for stopping drug therapies and adverse events possibly related to drug therapy were not collected, thus we cannot exclude bias due to therapy interruption because of side effects.

Finally, the possibility of unmeasured residual confounding cannot be completely ruled-out. However, the E-value for the lower boundary of the confidence interval for the detrimental association of DRV/c with death has the large value equal to 2.43, indicating that the confidence interval could be moved to include the null by a strong unmeasured confounder associated with both DRV/c treatment and death with a risk ratio of 2.43-fold for each, above and beyond all the measured confounders. Weaker confounders, however, could not do so.

## Conclusion

In conclusion, in a large cohort of patients with COVID-19 we found no association between LPV/r treatment and risk of death but an increased risk of death related to treatment with DRV/c. Although these data are not conclusive, the inappropriate use of this drug combination in the present pandemic entails the risk of shortage of a drug that is currently used as a second-line treatment for people with HIV.

## Data Availability Statement

The datasets presented in this article are not readily available because a consensus by the authors is needed. Requests to access the datasets should be directed to corresponding author.

## Ethics Statement

The study was approved by the institutional ethics board of all recruiting centers (*N* = 33). The ethics committee waived the requirement of written informed consent for participation.

## Author Contributions

LI and AD: had full access to all data in the study, took responsibility for the integrity of the data and the accuracy of the data analysis, drafting of the manuscript, and supervision. AD, LI, and RD: concept and design. AD, SC, AG, RA, GV, and GS: statistical analysis. All authors: acquisition, analysis, interpretation of data, critical revision of the manuscript for important intellectual content, administrative, technical, or material support.

## Conflict of Interest

The authors declare that the research was conducted in the absence of any commercial or financial relationships that could be construed as a potential conflict of interest.

## Appendix

### Clinical Centers

1. Centro Cardiologico Monzino IRCCS, Milano.

2. Humanitas Clinical and Research Hospital IRCCS, Rozzano-Milano.

3. IRCCS Policlinico San Donato, San Donato Milanese (MI).

4. ASST Milano Nord - Ospedale Edoardo Bassini. Cinisello Balsamo (MI).

5. Fondazione IRCCS Policlinico San Matteo, Pavia.

6. Ospedale di Circolo e Fondazione Macchi di Varese. Varese.

7. Ospedale San Gerardo, ASST Monza. Monza.

8. Ospedale di Cremona, Cremona.

9. Ospedale Maggiore della Carità. Novara.

10. Azienda Ospedaliera Universitaria di Padova. Padova.

11. Azienda Ospedaliero - Universitaria di Modena. Modena.

12. Ospedale Morgagni-Pierantoni Forlì.

13. Ospedale di Ravenna. AUSL della Romagna. Ravenna.

14. Azienda ospedaliero-universitaria Careggi. Firenze.

15. Azienda Ospedaliero-Universitaria Pisana. Pisa.

16. Azienda Sanitaria Locale (AUSL) di Pescara, Pescara.

17. Ospedale Clinicizzato SS. Annunziata. Chieti.

18. Istituto nazionale per le malattie infettive Lazzaro Spallanzani, IRCCS. Roma.

19. Fondazione Policlinico Universitario A. Gemelli IRCCS, Roma.

20. Columbus Clinic Center. Roma.

21. Fondazione I.R.C.C.S “Casa Sollievo della Sofferenza”, San Giovanni Rotondo, Foggia.

22. IRCCS Neuromed, Pozzilli (IS).

23. Azienda Ospedaliera Universitaria “Federico II”. Napoli.

24. Ospedale del Mare, ASL NA1. Napoli.

25. PO S. Maria di Loreto Nuovo -ASL Napoli 1 Centro. Napoli.

26. Azienda Ospedaliera dei Colli, Ospedale Cotugno, Napoli.

27. Ospedale di Boscotrecase - ASL Napoli 3. Napoli.

28. EE Ospedale Regionale F. Miulli, Acquaviva delle Fonti (BA).

29. P.O. San Giuseppe Moscati, Taranto.

30. Azienda Ospedaliero Universitaria Mater Domini. Catanzaro.

31. P.O. “San Marco”, AOU Policlinico-Vittorio Emanuele. Catania.

32. Azienda Ospedaliera Universitaria. Policlinico-Vittorio Emanuele. Catania.

33. Azienda Universitaria Policlinico Paolo Giaccone. Palermo.
